# Advances in Gas Detection of Pattern Recognition Algorithms for Chemiresistive Gas Sensor

**DOI:** 10.3390/ma17215190

**Published:** 2024-10-24

**Authors:** Guangying Zhou, Bingsheng Du, Jie Zhong, Le Chen, Yuyu Sun, Jia Yue, Minglang Zhang, Zourong Long, Tao Song, Bo Peng, Bin Tang, Yong He

**Affiliations:** 1 Chongqing Key Laboratory of Optical Fiber Sensor and Photoelectric Detection, Chongqing University of Technology, Chongqing 400054, China; guangyingzhou@stu.cqut.edu.cn (G.Z.); bingshengdu@cqut.edu.cn (B.D.); zhongjie@stu.cqut.edu.cn (J.Z.); leojesonchen@163.com (L.C.); syy18767865@stu.cqut.edu.cn (Y.S.); 18883090069@stu.cqut.edu.cn (J.Y.); 15023823322@163.com (M.Z.); longzourong@cqut.edu.cn (Z.L.); tsong@cqut.edu.cn (T.S.); 2 Key Laboratory of Optoelectronic Technology and Systems of the Education Ministry of China, College of Optoelectronic Engineering, Chongqing University, Chongqing 400044, China; yonghe@cqu.edu.cn

**Keywords:** chemiresistive sensors, selectivity, gas detection, pattern recognition, feature extraction

## Abstract

Gas detection and monitoring are critical to protect human health and safeguard the environment and ecosystems. Chemiresistive sensors are widely used in gas monitoring due to their ease of fabrication, high customizability, mechanical flexibility, and fast response time. However, with the rapid development of industrialization and technology, the main challenges faced by chemiresistive gas sensors are poor selectivity and insufficient anti-interference stability in complex application environments. In order to overcome these shortcomings of chemiresistive gas sensors, the pattern recognition method is emerging and is having a great impact in the field of sensing. In this review, we focus systematically on the advancements in the field of data processing methods for feature extraction, such as the methods of determining the characteristics of the original response curve, the curve fitting parameters, and the transform domain. Additionally, we emphasized the developments of traditional recognition algorithms and neural network algorithm in gas discrimination and analyzed the advantages through an extensive literature review. Lastly, we summarized the research on chemiresistive gas sensors and provided prospects for future development.

## 1. Introduction

With the rapid development of industrialization and the rise of big data, gas monitoring has been widely used in high-demand sectors such as environmental protection, medical health, military aerospace, life safety, and industrial production [1,2,3,4]. Accordingly, the necessary chemiresistive, electrochemical, optical and thermal conductivity gas sensors are constantly being researched and developed [5,6,7,8]. Among them, semiconductor gas sensors have become the most widely used gas sensors due to their small size, high sensitivity, low cost and simple manufacturing process [9,10,11,12,13,14]. Semiconductor gas sensors convert details of target gases into electrical signals based on the charge transfer that occurs during chemisorption between the target gases and the sensitive materials. However, these gas sensors generally suffer from poor selectivity due to the multiphase adsorption characteristics of sensitive materials [13,14]. Thus, it is necessary to develop high-selectivity gas sensors for reliable gas detection.

Many strategies of hybrid materials, single atom modification, and other cutting-edge technologies have enhanced the capabilities of these chemiresistive gas sensors, paving the way for highly sensitive and selective sensing [15,16,17,18,19,20]. However, these strategies generally acquire effective detection in single gases or simple gas environments, and the needs are still unsatisfied in complex gas environment detection such as distinguishing gases with similar chemical properties. Recently, an artificial olfactory system named E-nose, which consists of gas sensor arrays with pattern recognition, has been developed to improve the selectivity in complex gas environment detection due to the availability of multiple recognition parameters and excellent properties of anti-environment interference. For example, Li and Yu made an electronic nose composed of an array of SnO_2_/WO_3_ nanocomposite chemical resistance sensors and built a quantitative P-nearest neighbor backpropagation neural network (pN-BPNN) model based on the extracted response characteristics. The qualitative identification of hydrogen, ammonia, hydrogen–ammonia, ethanol, acetone, ethanol–acetone, toluene and formaldehyde and the quantitative estimation of gas concentration were realized [11].

Pattern recognition is considered to be an advantageous tool for the development of intelligent devices, which can effectively solve the selectivity problem, and has been applied to various complex environments such as breath and factory exhaust. In order to improve the efficiency of gas recognition, several classical feature extraction methods and recognition algorithms have been developed [21,22,23], including the data processing methods of curve fitting parameters and transform domain, and recognition algorithms of traditional recognition algorithms, artificial neural network (ANN) [12,13,24], convolutional neural network (CNN) [25], multi-layer perceptron (MLP) [26,27,28,29,30,31] and others. For example, Oh and Kim [32] used a sensor array consisting of five In_2_O_3_ semiconductor metal oxide (SMO) gases to detect and distinguish five indoor volatile organic compounds (VOCs), namely benzene, xylene, toluene, formaldehyde, and ethanol. By using three supervisory algorithms: artificial neural networks (ANN), deep neural networks (DNN), and one-dimensional convolutional neural networks (1D CNN), this machine learning-based approach successfully distinguished indoor pollutants even when ambient humidity and temperature changed. However, there are many kinds of noise and interference in the actual environment, such as ambient humidity, weak response signals, and similar molecular characteristics, which bring difficulties to the identification of target gases. It is significantly important to explore a suitable pattern recognition algorithm for gas recognition in real environments [33,34,35,36]. For example, traditional pattern recognition algorithms perform well on small data sets, are easy to understand and have low computational costs; artificial neural network algorithms show good noise resistance and better handle large data sets [37,38,39]; while MLP is capable of effectively modeling nonlinear relationships in data. Therefore, it is necessary to update and comprehensively evaluate the existing pattern recognition algorithm to improve the accuracy of gas recognition [29,40,41,42].

In this review, we systematically describe the research progress of gas sensing pattern recognition algorithms. The first part of this review provides a description of the evolution of chemiresistive sensors and the meaning of the pattern recognition algorithms for gas sensing. The Section 2 introduces the performance evaluation of recognition algorithms in gas sensing. The Section 3 focuses on the data preprocessing and feature extraction methods in gas sensor arrays in recent years. In the Section 4 and Section 5, the research results of traditional pattern recognition algorithms and neural network algorithms for gas detection are summarized and provide insights into the merits of the various pattern recognition algorithms. Finally, the challenges and prospects of pattern recognition in gas sensing applications are presented.

## 2. Performance Evaluation

In the general data training process for gas sensing, the loss function is crafted from label values and algorithm predictions, optimizing the model parameters to minimize errors. To avoid overfitting and ensure the generalization ability of the model, cross-validation can be employed. For example, K-fold cross-validation divides the data set into k non-overlapping subsets, takes one subset in turn as a test set and the rest as a training set for model training and evaluation, and takes an average performance after repeating k times to ensure that the model can be stably and effectively generalized to previously unseen data.

After training is completed, the model’s generalization is evaluated on a dedicated test set. Algorithm selection is crucial for recognition outcomes [23,40]. Performance metrics of accuracy, recall, specificity, and F1 score are employed to gauge generalization.

Accuracy reflects the proportion of correctly classified samples, given by (TP + TN)/(TP + FP + TN + FN), where TP = true positive, TN = true negative, FP = false positive, and FN = false negative. Recall, or true positive rate, assesses positive instance detection, formulated as TP/(TP + FN). Higher recall indicates more positive samples detected. Specificity, or true negative rate, quantifies negative instance identification, calculated as TN/(TN + FP). F1 score, the harmonic mean of precision and recall, balances the two, calculated as 2 × (Precision × Recall)/(Precision + Recall), where Precision = TP/(TP + FP).

To handle overfitting, bootstrapping can be used. Bootstrapping creates multiple training and test sets through multiple random samples with resampling, repeatedly training and evaluating the model to ensure that the model can be stably and accurately generalized to previously unseen data.

## 3. Data Pre-Process Methods

The accuracy of gas recognition depends on the characteristics of the data, such as response, sensitivity, response time, and reaction kinetics parameters, so it is necessary to optimize the raw data by preprocessing and feature extraction, remove redundant information, and extract distinguishable steady-state and transient features to improve recognition accuracy. These processing methods of feature extraction play an important role to improving the effectiveness of pattern recognition algorithms, especially in dealing with noise, errors, and sensor drift to ensure accurate identification of different types and concentrations of gases [43].

Feature extraction includes extracting key signal segments from the sensor data and analyzing their characteristics on the response curve, such as peak value, integration, rate of change, and slope of adsorption rate in a specific interval [44]. Additionally, time-frequency conversion techniques, such as DWT [31,45,46], FFT [16,27,33], and so on, can effectively capture complex patterns and nonlinear features in gas sensor sensing signal. In the next part, the feature extraction methods are summarized as follows. 

### 3.1. Drift Compensation

Sensor drift is an issue that arises in sensor arrays due to the influence of factors such as temperature fluctuations, changes in humidity, degradation over time, and varying pressure. In complex application scenarios, interference persists, making drift a major challenge for gas sensor technology, damaging its detection accuracy and hindering the widespread application of electronic nose systems [44]. In the last 5 years, researchers have developed a range of drift-resistant models aimed at addressing this persistent issue.

Heng et al. [9] proposed a semi-supervised adversary-domain adaptive convolutional neural network (SAD) method, which combined with the maximum mean difference and minimum variance constraints of regenerated Hilbert space, effectively solved the long-term drift problem of electronic nose and device displacement and showed better classification accuracy than other methods in the experiment. The classification accuracy of different scenes in the long-term drift data set were 78.01% and 82.53%, respectively, and the classification accuracy of the instrument change data set were 96.45%. Sun et al. [10] proposed an unsupervised domain adaptive framework that utilizes transformer based dynamic encoder and prototype learning to realize sensor drift compensation, which significantly improved the average accuracy by 11.12% and reached the highest average accuracy of 92.67% on the CQU dataset. Both Li et al. [11] and Zhang et al. [12] applied a relative method based on the ratio of baseline to steady-state resistance to reduce drift during the pretreatment process. Aurora, A. [20] proposed a new method combining the auxiliary sensor response with the self-calibration algorithm, which significantly improved the accuracy of the Figaro TGS2600 module under the temperature range of −10 °C to 40 °C and the relative humidity conditions of 35%, 65% and 95%. Carbon monoxide tests show sensor accuracy improved by a factor of up to 11 and error reduced to about ±0.8 ppm CO. Krivetskiy et al. [45] propose a novel approach to preprocessing the response of temperature-modulated metal oxide gas sensors using a statistical signal shape analysis (SSA) algorithm, aiming to improve the discrimination of closely related gases under realistic atmospheric conditions through machine learning (ML) algorithms. At the same time, the effects of sensor response drift and amplitude fluctuation were eliminated more effectively.

### 3.2. Denoising Processing

Noise is usually caused by measurement errors, environmental interference, or distortion in data transmission, which can make the data chaotic and difficult to extract useful information. Therefore, before extracting features, it is necessary to perform appropriate denoising on the data to reduce or eliminate these random errors. The main methods for denoising include applying filters, smoothing algorithms, statistical methods, and machine learning techniques.

Wang et al. [47] proposed an optimally designed FIR complementary filter, which achieves a wider low frequency bandwidth by solving the optimal coefficients through convex optimization problems and has better performance than traditional IIR complementary filters. Golmohammadi et al. [48] used the strain data collected by the FBG sensor to study and optimize the discrete wavelet transform parameters for effective denoising, and successfully recovered the low-amplitude strain signal without losing any data. Guo et al. [49] proposed and verified a method to suppress the low frequency noise of vector magnetic sensors by rotating modulated signals, effectively modulating low frequency signals to a high frequency with demodulation to reduce noise. Harindranath and Arora [50] developed a simulation platform to predict algorithm performance by experimentally measuring the noise characteristics of commercial IMU and compared the old and new algorithms in simulation and actual experiments, showing that the angle error of the new algorithm on low-cost IMU was <1. Wang et al. [51] used a transformer-based U-channel network to optimize photoacoustic signal processing to reduce background noise, achieving a HCN detection limit of 0.89 ppb and a 1 s response time, and experimentally verified its high-sensitivity real-time monitoring capability in environmental monitoring and industrial safety applications. 

### 3.3. Original Response Curves

**Figure 1:**
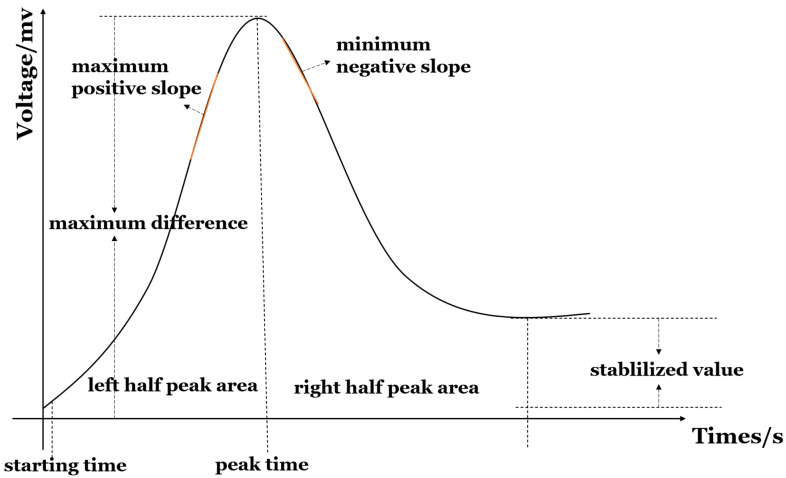
Features of original response curve.

Extracting segmented signal features of the sensor’s original response curve is a common feature extraction method. These characteristics include a steady-state response and a transient response. Generally, those features include maximum value, integral, derivative, area value, rise time, fall time, rise slope, fall slope, maximum difference, relative maximum, fractional maximum, logarithmic maximum, derivative, and integral [52], as shown in Figure 1. The maximum value represents the final steady-state characteristic of the entire dynamic response process and reflects the maximum change in the sensor’s response to odor, which is often used as one of the simplest and most common features in electronic noses. Moreover, the parameters of response time, recovery time, rise slope and fall slope reflect the kinetic reaction process of gas sensing, which is significant to improve the accuracy in rapid gas identification. Recently, Li and Yu [11] prepared PD-doped WO_3_ and PD-doped SnO_2_ sensor arrays and extracted the parameters of response time, recovery time and resistance variation of 15 ppm hydrogen at different temperatures, realizing the gas detection. Deng et al. [53] reported a method of extraction parameters from original and light modulation data (100 ppm ethanol) of a PT-modified zinc oxide gas sensor to detect 100 ppm ethanol. Acharyya et al. [27] used a tin oxide hollow ball gas sensor to obtain the transient response curves of formaldehyde, methanol, propyl alcohol and toluene gases, which showed the corresponding responses, providing information for subsequent DWT feature extraction. Chu et al. [54] used a sensor array consisting of 4 sensors to extract 12 features, such as response value, response time and recovery time, from each sample. By extracting features (response value, response and recovery time) from the dynamic curve, two additional features of the sensor were also extracted, namely response time (τres) and recovery time (τrec), acquiring ultra-high discrimination accuracy. 

### 3.4. Curve Fitting Parameters

Another method is to fit the sensor response curve or the response parameters and then extract features based on the model coefficient. Curve fitting is a data processing method that approximates the function relationship of discrete points by using empirical equation or analogies [55]. However, in curve fitting, selecting and assuming the concrete form of the unknown function is still a key and complex problem.

**Figure 2:**
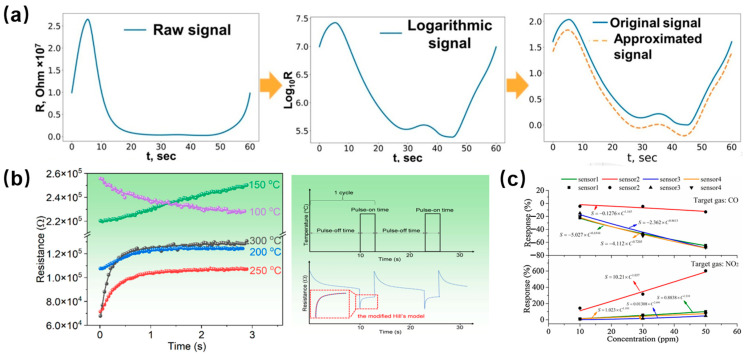
(**a**) Signal preprocessing and polynomial curve fitting. Reprinted with permission from Ref. [45]. Copyright 2021, Elsevier. (**b**) Fitting the corresponding transient resistances of ethanol, formaldehyde, toluene and acetone gases to the modified Hill equation. Reprinted with permission from Ref. [56]. Copyright 2023, Elsevier. (**c**) Steady-state response and fitting curves for CO and NO_2_. Reprinted with permission from Ref. [54]. Copyright 2020, Sensors.

As shown in Figure 2a, Krivetskiy et al. [45] proposed an approach based on PCF polynomial curve fitting to extract the characteristics of the data. This method improves the selectivity for low concentration VOCs with similar chemical properties. Recently, Niu et al. [56] reported a method for calibration and identification of gas sensors by using the temperature pulse modulation method. In the pulse period, the modified Hill equation was used to calibrate the transient resistance of the sensor, and then the relevant parameters were extracted from the modified Hill equation to detect various gases. Moreover, Chu et al. [54] used an array composed of four sensors to realize CO and NO_2_ gas detection by extracting the feature parameters of the fitting plots of the response time and recovery time. 

### 3.5. Transform Domain

The transform domain method distinguishes the material characteristics by the transform coefficient. Fourier transform, as the cornerstone of signal processing, converts signals from the time domain to the frequency domain, and FFT optimizes the discrete signal processing to reduce computing costs. However, the delocalization of the Fourier transform information limits the acquisition of time information. To this end, STFT tries to introduce a time dimension, but is still constrained by the window length. Furthermore, wavelet transform, especially DWT, with its unique adaptive window width technology, can deeply analyze frequency components while maintaining time information and is especially good at capturing abrupt features in signals, becoming the preferred tool for VOCs feature extraction [16]. Moreover, DWT can accurately capture local frequency information through the flexible transformation of wavelet basis functions, such as extension and translation, which greatly enhances its applicability and flexibility.

Meng et al. [57] adopted fourth-order wavelet transform and SVM mode to identify ethanol, methanol, propanol, 2-butanone and butyl acetate gases, and the accuracy rate was increased from 92.24% to 99%. Acharyya et al. [46] proposed a DNN and DWT model to reduce the average error of acetone, benzene, ethanol, formaldehyde, methanol, propanol and toluene gases to 8.41%, 9.68%, 7.23%, 6.85%, 5.33%, 6.57% and 8.11%, respectively. Acharyya et al. [31] applied discrete wavelet transform (DWT) technology to the transient response curve to extract features from the signal, resulting in an average accuracy of 93.75%, 94.67%, 92.3% and 91.45% for the identification of formaldehyde, methanol, propanol and toluene gases, respectively. Acharyya et al. [27] used fast Fourier transform (FFT) and discrete wavelet transform (DWT) for global normalization to achieve excellent feature extraction. 

## 4. Traditional Recognition Algorithms 

Machine learning is an effective data processing method for extraction parameters that has been extensively applied to improve selectivity and compensate for the error of gas detection [58]. These methods are multidimensional in the analysis of feature parameters, and the common methods mainly include principal component analysis (PCA), LDA, and support vector machine (SVM), KNN and random forest. The main advantage of these methods is their ability to process large amounts of data and simplified information for the end user. Here, the traditional recognition algorithms are systematically introduced for their application in gas recognition.

### 4.1. Principal Component Analysis

Principal component analysis (PCA), also known as matrix data analysis, is a common linear dimensionality reduction technique. This method is generally applied to discover and develop relationships between different variables in a large multivariate data set and condense them into lower-dimensional space, simplifying the multivariate data set [40].

**Figure 3:**
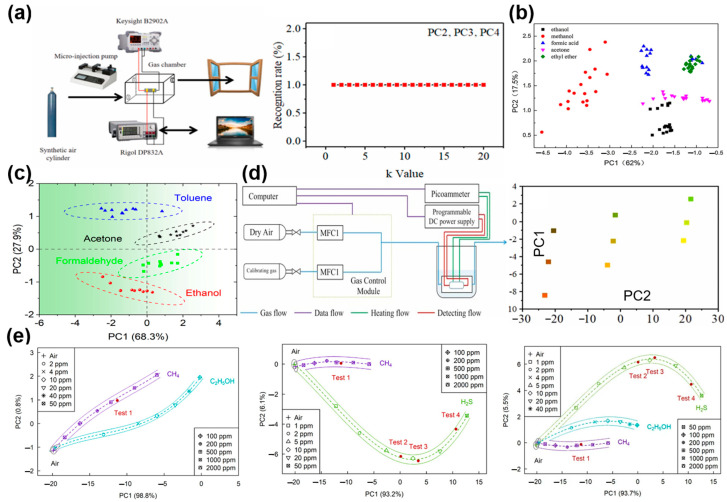
(**a**) SnO_2_ sensor platform for identification of medicinal chemicals and PCA identification results. Reprinted with permission from Ref. [60]. Copyright 2021, Elsevier. (**b**) PCA classification results for ethanol, methanol, acetone, formic acid and ether. Reprinted with permission from Ref. [53]. Copyright 2017, Elsevier. (**c**) PCA classification results for ethanol, formaldehyde, toluene and acetone. Reprinted with permission from Ref. [56]. Copyright 2024, Elsevier. (**d**) The general block diagram of ether recognition by an electronic nose system and the distribution diagram on the two-dimensional plane after PCA dimensionality reduction [61]. Copyright 2023, Elsevier. (**e**) Principal component analysis results for partial hydrogen corresponding to air, methane and ethanol; principal component analysis results corresponding to air, hydrogen sulfide and methane; principal component analysis results corresponding to air, hydrogen sulfide, methane and ethanol. Reprinted with permission from Ref. [55]. Copyright 2023, Elsevier.

In recent years, the PCA algorithm has been widely used in the research of gas classification and recognition in environmental monitoring, public safety and other fields, which is summarized in Table 1. For instance, Meng et al. [59] built a complete sensor test platform, analyzed the recognition rates of precursor chemicals using principal component analysis, and carried out quantitative evaluation using SVR. The results showed that the MAE of dynamic measurement was better than that of static measurement, and the accuracy of dynamic identification of interference samples could reach 87.5%. As shown in Figure 3a, the drug detection platform consists of an air chamber, a synthetic gas cylinder, a microjet pump, a programmable DC power supply, and a computer. Ji et al. [60] used principal component analysis (PCA) without PC1, and the recognition rate was 100% regardless of the k value used. Deng et al. [53] proposed a PT-modified ZnO gas sensor to distinguish ethanol, methanol, acetone, formic acid and ether gases based on a temperature and light modulation method. The principal component analysis method was used to analyze the features extracted from the response curves, and the recognition accuracy reached 95.55%; the visualization results are shown in Figure 3b. In addition, Niu et al. [56] used a temperature pulse operation strategy to detect low concentrations of VOCs, and successfully used PCA combined with the Hill equation to increase the recognition rate to 100% at a 500 ppb concentration of ethanol, formaldehyde, toluene, acetone and other gases (Figure 3c). As shown in Figure 3d, Ji et al. [61] proposed a self-made gas-sensitive measurement method for ether gas, obtained dynamic response signals through temperature modulation, and improved the recognition accuracy of 0~5 V, 1~5 V, and 2~5 V triangular waves from 83.33%, 90%, and 96.66% to 100% through PCA inverse transformation. As shown in Figure 3e, Shaposhnik et al. [55] proposed a single gas sensor that used a self-made gas sensing layer to selectively distinguish between hydrogen sulfide, ethanol, and methane using the PCA algorithm, with an accuracy of 99.3%.

Although PCA has shown advantages in unsupervised learning and linear data processing, it is ineffective in differentiating electronic nose datasets that contain classification information and nonlinear features. This limitation largely restricts the wide application and development of chemiresistive gas sensors.

### 4.2. Linear Discriminant Analysis

LDA, as a multidimensional data analysis strategy, is similar to PCA, but their purposes and implementation methods are different. LDA is committed to characterizing datasets through well-designed orthogonal dimensions that not only measure the dispersion of the data, but also incorporate considerations of statistical properties, such as mean values. Unlike PCA, which simply seeks to maximize the direction of data variation, LDA pays more attention to using the power of statistical analysis to accurately classify each data instance into its category [44]. It is precisely because of its unique dimension selection mechanism that LDA shows better classification performance than PCA after simplifying the data to low-dimensional space.

**Figure 4:**
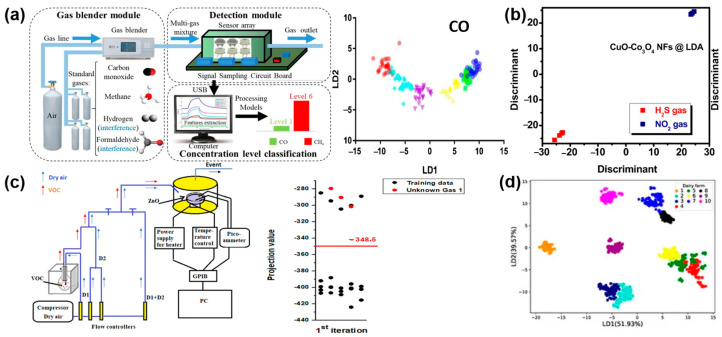
(**a**) Identify the overall block diagram of the electronic nose system for CO and CH_4_ and the LDA differentiation results. Reprinted with permission from Ref. [12]. Copyright 2021, Elsevier. (**b**) LDA differentiation results of H_2_S and NO_2_ gases. Reprinted with permission from Ref. [67]. Copyright 2023, Elsevier. (**c**) Experimental apparatus for the detection of ethanol, acetone, isopropyl alcohol, methanol and toluene and classification results of LDA. Reprinted with permission from Ref. [68]. Copyright 2023, RSC advances. (**d**) LDA differentiation results of milk samples from different farms. Reprinted with permission from Ref. [69]. Copyright 2020, Sensors.

For example, Swarga et al. [66] proposed an identification system to measure tobacco by using the LDA algorithm, acquiring an average recognition accuracy of 93%. In the field of gas detection, as shown in Figure 4a, Zhang et al. [12] fabricated an electronic nose system and constructed a gas identification model based on the LDA algorithm, obtaining a high accuracy of 89.07% and 92.35% for the gases of carbon monoxide and methane under the interference of hydrogen and formaldehyde. As shown in Figure 4b, Phuoc et al. [67] proposed four kinds of NFs-based sensors and exhibited high selectivity for H_2_S and NO_2_ in the low concentration of 0.1–1 ppm based on the LDA algorithm (Figure 4b). As shown in Figure 4c, Souissi et al. [68] used ZnO-based sensors to detect the sensing process of mixed gas and also used the LDA algorithm to detect ethanol and toluene efficiently. As shown in Figure 4d, Mu et al. [69] developed an electronic nose composed of seven metal oxide semiconductor sensors to realize rapid classification of milk samples through reduction and feature fusion through LDA technology.

However, it is worth noting that the LDA method may cause overfitting problems due to excessive dependence on data, which will affect the accuracy of gas identification and make its performance decline.

### 4.3. Support Vector Machines

Support vector machine (SVM) is an optimization model based on a mathematical mapping mechanism whose purpose is to accurately classify data sets. The model implements binary partitioning of data by mapping it to a specific segment plane (hyperplane) in a high-dimensional space [41]. In addition, SVM has introduced nonlinear variants to enhance their applicability in complex scenarios. Due to its characteristics of single processing and iterative learning, SVM can efficiently process limited data sets, which is different from LDA, PCA and other methods that require large training samples to show advantages. SVM makes full use of each data point in the iterative process, getting rid of the dependence on covariance information and maintaining stable prediction accuracy even on small-scale data sets, which is more flexible and efficient than LDA and PCA.

Recently, Mu et al. [69] developed an electronic nose consisting of seven MOS gas sensors to establish a classification model for milk source identification by using three machine learning algorithms: logistic regression (LR), support vector machine (SVM) and random forest (RF). The results showed that the SVM model based on LDA fusion features demonstrated the best performance, with an accuracy of 95%. In another study, Kanaparthi et al. [70] applied SVM to process data composed of sensor response and ternary logic to predict the presence of gases in the air, and it exhibited high prediction accuracy. Meng et al. [71] tested the dynamic output current of a sensor for gases with different concentrations (such as ethanol, toluene, 2-butanone, acetone and ether), and acquired a classification accuracy of 100% based on the SVM algorithm. Moreover, Thai and Tonezzer et al. [72] chose the support vector machine (SVM) to construct classification models for gas recognition. Their results proved that the SVM algorithm can distinguish all of the tested gases well, and the classification performance in 4D space was better than that in 2D space.

SVM, as a small sample learning technology, has the unique advantage of being able to efficiently handle the small-scale data sets of an electronic nose. However, in a case of missing data, the performance of this method can be severely affected, which may reduce the accuracy of gas identification.

### 4.4. Random Forest

Random forest (RF) algorithms perform learning and prediction tasks of data by integrating multiple decision trees. Specifically, the algorithm builds a collection of decision trees, and each of them learns and predicts independently from the input sample. Ultimately, RF determines the final category of the sample by aggregating the predictions of these decision trees, usually using a majority voting mechanism [73].

Sun et al. [74] used common machine learning algorithms of RF and KNN to build an intelligent gas sensing mode, and this sensing mode has been successfully applied in cigarette brand recognition. Both of these machine learning algorithms have a high accuracy rate, more than 95%; specifically, KNN has an accuracy rate of 96.29%, while RF has a slightly better accuracy rate of 96.32%. Acharyya et al. [28] used SnO_2_ gas sensors to identify formaldehyde, 2-propanol, toluene, and methanol. After comparing different machine learning algorithms, including RF, SNM, NB, MLP, etc., it was found that RF showed the best performance, with a maximum accuracy of 100% and an average accuracy of 85.93%. Mu et al. [69] developed an electronic nose consisting of seven MOS sensors to identify the source of milk and estimate the fat and protein content of milk. The recognition model was constructed by using GBDT, XGBoost and RF algorithms, and the results showed that the RF algorithm exhibited the best performance (milk fat R^2^ = 0.9399; milk protein R^2^ = 0.9301).

RF (random forest) shows significant advantages in dealing with high dimensional and unevenly distributed data sets. However, when facing a small sample size data set, its classification performance may be limited. In addition, compared with a single decision tree algorithm, the construction process of a RF model is complex, which leads to an increase in computing consumption.

### 4.5. K-Nearest Neighbor

The K-nearest neighbor (K-NN) algorithm is a widely used technique in the field of supervised learning, suitable for both classification and regression tasks. The core mechanism is training the data set to construct a partitioning strategy for the feature space, which is then solidified into an algorithmic model that can be used to classify new samples or make regression predictions [35].

Recently, Li and Yu [11] proposed a thin conical metal housing excited by a piezoelectric disk to serve as a focused ultrasonic transducer and protective housing for the SnO_2_ gas sensor, innovatively integrating two gas sensing units on the metal halide perovskite substrate to create a compact and efficient gas sensor array. Then, the recognition mechanism was constructed by using the K-nearest neighbor (KNN) algorithm, which realized accurate classification of eight gases and their mixtures, such as hydrogen and ammonia, with a recognition rate up to 99.86%. In another study, Xu et al. [75] proposed a MOS gas sensor array to construct a new hybrid method for gas identification. The method classifies the gas by the K-nearest neighbor (KNN) algorithm and detects the concentration by MVRVM regression. The experimental results showed that the accuracy was 98.33%, which is better than the PCA and ICA algorithms. 

The K-NN algorithm is widely used in the field of gas identification, and its popularity is mainly due to its simplicity and good adaptability to the diversity of data distribution. Nevertheless, K-NN may exhibit high computational complexity when dealing with large data sets and be sensitive to noise and interference with non-critical features that are common in gas sensor data.

## 5. Neural Network Algorithm

In the previous chapter, we introduced the application and progress of traditional recognition algorithms in the field of gas sensing. Although these traditional recognition algorithms exhibit some advantages in gas detection, there are obvious shortcomings in complex environment gas detection. To improve the accuracy of pattern recognition, a neural network algorithm was developed to closely match the distribution of feature spatial data, reduce redundant information, and ensure the efficiency of gas detection. In the following part, neural network algorithms are introduced.

### 5.1. Artificial Neural Network (ANN)

Artificial neural networks (ANN), as an information processing tool, mimic the way biological brains work, and unlike traditional computer algorithms that execute instructions, they rely on highly interconnected networks of neurons to process information in parallel. The ANN structure usually includes an input layer, a hidden layer, and an output layer [76]. The input layer receives data from the gas sensor’s signal, and the output layer feeds back the processing results to the user through the machine control system. The neurons in the hidden layer are responsible for processing the input data, and these data are transferred to the output layer through a specific function. The connection mode of the neurons determines the data processing strategy, and the user can adjust these connections based on the features and application requirements of gas detection.

**Figure 5:**
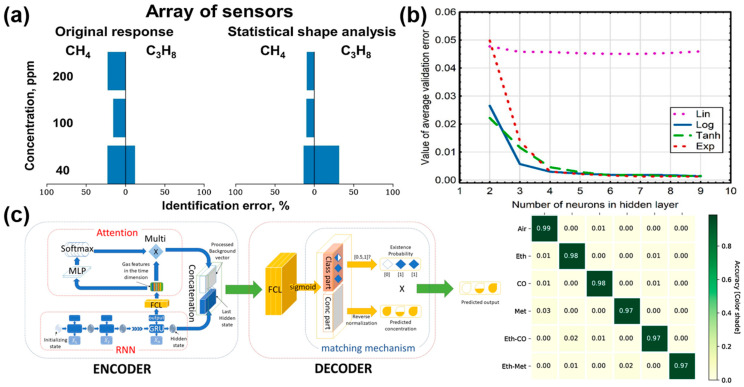
(**a**) A concentration-dependent analysis of the identification scores of methane and propane error gases was performed using an artificial neural network (ANN)-based algorithm. Reprinted with permission from Ref. [45]. Copyright 2021, Elsevier. (**b**) The number of neuronal nodes in the hidden layer and the type of activation function have an impact on the results in terms of the mean value of the validation error describing the acetone and ethanol levels in the analyzed mixture. Reprinted with permission from Ref. [77]. Copyright 2022, Sensors. (**c**) Hierarchy structure and confusion matrix of 2L-ARNN algorithm for monitoring mixed gases and corresponding concentration estimation. Reprinted with permission from Ref. [78]. Copyright 2021, Elsevier.

As shown in Figure 5a, Krivetskiy et al. [45] used three SnO_2_-based materials: pure SnO_2_, gold modified SnO_2_, and bimetallic Au and Pd modified SnO_2_ to construct a sensor array. A machine learning algorithm based on an artificial neural network (ANN) was used to preprocess the signals from a temperature-regulated array sensor through a statistical shape space, thereby greatly improving the selectivity of gas recognition. Moreover, Wawrzyniak [77] proposed a method based on B-spline curves and an artificial neural network (ANN) to detect gases via the thermal modulation MOS gas sensor of TGS2610C and realized quantitative analysis of volatile components (ethanol and acetone) in a mixture, as shown in Figure 5b. 

ANN training is mainly realized through two ways: supervised learning and unsupervised learning. In the gas detection process, the operation of an E-nose mainly relies on supervised learning, which requires a large number of labels to guide the network learning to construct the identification model. Although unsupervised learning has many advantages in terms of autonomy, it is still in its infancy for gas detection at present.

### 5.2. Recurrent Neural Network (RNN)

RNN introduces a memory mechanism based on traditional neural networks to save historical information and output it by combining it with the current input. This design enhances the sensitivity of the RNN to time series data [26].

Schober et al. [79] detected low O_3_ and NO_2_ based on a graphene gas sensor, built an identification model using the RNN algorithm, and obtained a low RMSE of 11.14 ppb and MAE of 9.87~10.95 ppb. As shown in Figure 5c, Pan et al. [78] adopted the double loss function recurrent neural network (2L-ARNN) algorithm to accurately recognize air, CO, ethylene, and methane within 5 s, with an accuracy of 97.67%, which is significantly improved compared with the traditional method. 

This RNN algorithm is able to efficiently extract key features in a gas sensing signal even with relatively small sizes of data, making it a popular choice in the field of rapid gas identification. However, the application of RNN should also consider the potential gradient dissipation problem, which may affect the training quality and final performance of the model.

### 5.3. Back Propagation Neural Network (BPNN)

BPNN, or a backpropagation neural network, is a multi-layered neural network model that combines forward propagation and error feedback. The network achieves complex nonlinear transformation from the input data to the desired output by finely adjusting the connection weights between the neurons in each layer [58]. In particular, it uses a backpropagation algorithm as a learning mechanism to effectively optimize these weights, allowing the network to predict or classify data with more accuracy.

**Figure 6:**
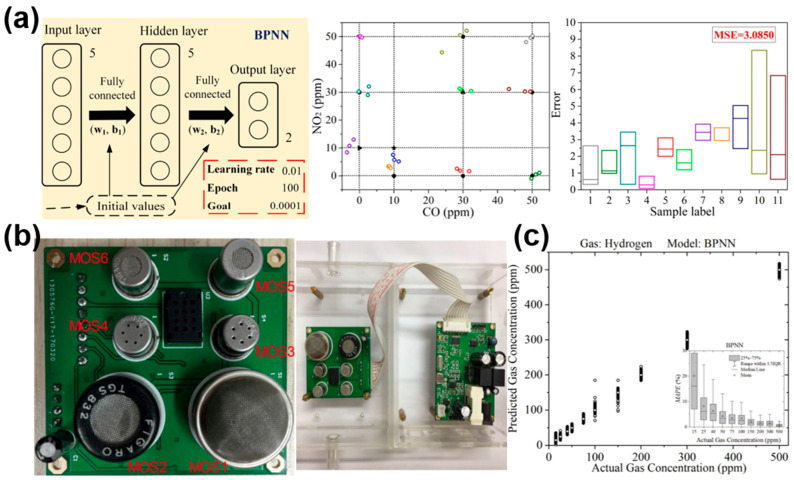
(**a**) BPNN models for the detection of various mixtures of CO and NO_2_. BPNN quantitative recognition performance. The error between the predicted BPNN concentration and the actual concentration is represented by the box plot. Reprinted with permission from Ref. [54]. Copyright 2021, Elsevier. (**b**) A prototype of an electronic nose based on MOS for detecting the odor of different wines. Reprinted with permission from Ref. [80]. Copyright 2018, Sensors. (**c**) No outliers are shown on the BPNN boxplot for each hydrogen concentration in the mixed gas. Reprinted with permission from Ref. [11]. Copyright 2023, Elsevier.

Chu et al. [54] used sensor arrays and backpropagation neural networks (BPNN) to identify 11 mixtures of nitrogen dioxide (NO_2_) and carbon monoxide (CO) in the concentrations of 0–50 ppm, exhibiting a high classification accuracy of 100%. Figure 6a shows the three-layer BP neural network, the quantitative recognition of CO and NO_2_ concentrations by BPNN, and the error of the test set. Liu et al. [80] adopted four machine learning technologies of XGBoost, RF, SVM and BPNN to construct a recognition mode distinguishing wine odors based on the portable electronic monitoring device of a TGS MOS gas sensor (in Figure 6b). The results showed that BPNN performs best in appellation and variety recognition, with an accuracy of 94% and 92.5%, respectively. Li et al. [11] established a P-neighborhood backpropagation neural network (pN-BPNN) model for quantitative estimation of concentrations. As can be observed in Figure 6c, the results showed that the average absolute percentage error of hydrogen detection in the pN-BPNN model decreased from 5.44% to 2.08% in the range of 15 to 500 ppm.

After a thorough review of the latest progress with the BPNN algorithm in the application of a chemiresistive-based electronic nose, we found that it exhibits excellent classification accuracy. However, the BPNN algorithm inevitably leads to a surge in computation when dealing with complex gas classification tasks, extending the training period. In order to improve the efficiency of a BP neural network in gas detection, it is necessary to optimize the network layer number and parameter configuration.

### 5.4. Radial Basis Function Neural Networks (RBFNN)

As a special feedforward network, RBFNN is characterized by its excellent approximation ability, simplicity in design, strong generalization potential, effective resistance to input noise, and excellent online learning adaptability [81]. Moreover, RBFNN can simulate any continuous nonlinear network mapping with high accuracy.

Jiang et al. [82] innovatively proposed the EQBC-RBFNN technology, inspired by the integration of RBFNN, to evaluate the performance of an electronic nose equipped with a SnO_2_-based MOS gas sensor for detecting benzene, toluene, formaldehyde and other indoor pollutants. Compared with LDA, BPNN, RBFNN, KNN, SVM and other traditional methods, this EQBC-RBFNN technology significantly improved the accuracy of the electronic nose in gas classification.

In the application of a gas sensor, the RBFNN algorithm significantly avoids the local optimal solution dilemma that BPNN may encounter, and its excellent robustness and real-time learning ability provide strong support for improving the overall performance of the sensor. However, it is worth noting that both RBFNN and BPNN share a common challenge regarding data requirements, namely that both algorithms require large amounts of data to adequately train network parameters. Therefore, exploring how to streamline the network architecture to achieve efficient training with less data and shorter time has become the focus of current research.

### 5.5. Convolutional Neural Network (CNN)

The convolutional neural network (CNN) is inspired by the workings of biological visual systems in nature. The network structure usually consists of one or more layers of convolution units, pooling modules and fully connected layers, and a combination of these layers can achieve efficient extraction and recognition of image features [37].

**Figure 7:**
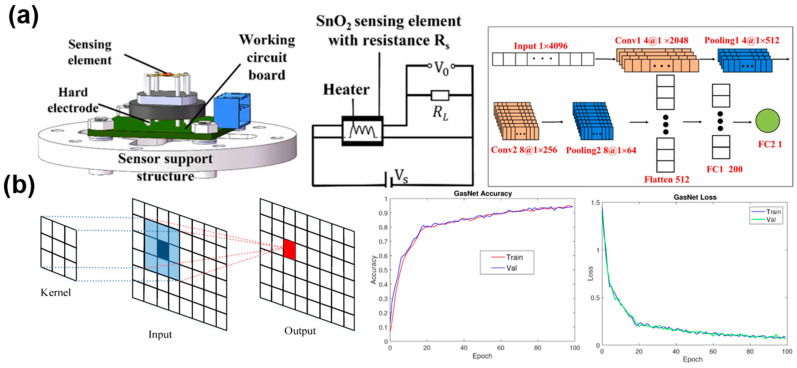
(**a**) Structure of SnO_2_ gas sensor unit and CNN model. Reprinted with permission from Ref. [26]. Copyright 2023, Elsevier; (**b**) MCNA framework for gas identification, training accuracy, validation accuracy, training loss, and validation loss. Reprinted with permission from Ref. [30]. Copyright 2017, Sensors.

Wang et al. [83] proposed an innovative joint recognition algorithm, GUCNN-XGBoost, in which the CNN structure is combined with a GRU unit, cascaded fusion BMC, and TSC. In the concentration range of 50~300 ppm, the identification accuracy of toluene, butanone and ether were improved to 97.54%. Pan et al. [84] introduced a transformer network to identify gases, combined a multi-scale convolutional network with a self-attention mechanism, and proposed a multi-scale convolutional neural network (MCNA) to identify nine gases such as acetaldehyde and acetone, achieving a high recognition accuracy of about 95%. Chu et al. [54] converted dynamic curves into grayscale images through grayscale processing and introduced a convolutional neural network (CNN) to construct a recognition model with an ultra-high recognition accuracy of 100%. Moreover, Yang et al. [26] used a SnO_2_ gas sensor to distinguish a variety of mixed gases based on the CNN model, with a detailed diagram of the sensor unit and the structure of the CNN model given in Figure 7a. The experimental results showed that it acquired a low prediction error of 12.3% in a low concentration (<1 ppm) and 5.7% in a high concentration (>1 ppm). In another study, Peng et al. [30] proposed a new 38-layer deep convolutional neural network (DCNN) for the detection of carbon monoxide, methane, hydrogen, and acetylene by using a gas sensor array composed of eight commercial MOS sensors. Figure 7b shows a structural framework that convolves two typical convolutional layers into a single convolutional block, as well as training accuracy and validation accuracy graphs, training loss graphs, and validation loss graphs. The experimental results showed that DCNN has higher classification accuracy than similar support vector machines (SVM) and multi-layer perceptrons (MLPs). 

The CNN algorithm shows excellent feature extraction ability in the application of gas sensing for sensor arrays and exhibits excellent classification accuracy and training efficiency. However, the selection of pooling layer parameters is very important for the classification results and it is easy to fall into local optimization during gradient descent training.

### 5.6. Summary of Gas Recognition Technology Based on Neural Networks

In Table 2, we summarized the research on semiconductor gas sensing applied to recognize gases using pattern recognition algorithms. It can be observed that a semiconductor gas sensor has been used to recognize VOCs, H_2_, NH_3_ and CO in the fields of environmental monitoring, public safety and food industry based on pattern recognition algorithms and realized ultra-high accuracy.

### 5.7. Comparison of Characteristics of Pattern Recognition Algorithms

In Table 3, we compare the characteristics of traditional algorithms and neural networks in gas pattern recognition. The traditional algorithm is fast in training and good in interpretation, but it is sensitive to losing data and suitable for classification and dimensionality reduction. Among them, LDA and SVM are suitable for classification tasks, PCA is used for dimensionality reduction, RF and K-NN perform well on large data sets, but K-NN is more complex for calculation. Neural networks are mostly supervised learning, which may be slow in training and require a large amount of data and regularization technology, but they have strong fault tolerance. RNN is good at sequence processing, CNN is good at image recognition, and both have good noise robustness. RBFNN and CNN train faster. Although the neural network model is complex and easy to overfit, with enough training samples, its accuracy is usually higher than the traditional algorithm, and it can achieve a higher recognition effect by adjusting the network structure and other optimization.

## 6. Summary and Outlook

This review demonstrates the key role of chemiresistive gas sensors in the field of gas detection, and discusses the challenges faced by sensors and the technological progress in recent years. Sensor selectivity and long-term stability are the focus of current research, and the application of advanced technologies such as nanotechnology, hybrid materials, and machine learning have significantly improved sensor performance. At the same time, response signal analysis, feature extraction, pattern recognition and other methods are also widely used. These methods, through more in-depth analysis and processing of sensor signals, effectively realize the compensation of drift, thus greatly improving the processing and interpretation of sensor data. These advances provide new possibilities for the development and application of gas detection technology. In the future, with the continuous advancement of science and technology, chemiresistive gas sensors need to make greater breakthroughs in performance and application. Regarding this, there are some suggestions that can be used to improve gas detection performance:

(1) The selectivity and stability of the sensor can be further studied and optimized by designing the structure of the gas sensor and testing models to meet actual needs in complex environments. For example, the FET type and the impedance type gas sensor can acquire more features than traditional resistive type gas sensors. Moreover, the pulse testing method can be an effective way to significantly enhance their sensing performance, such as pulse heat and pulse light modulation methods, which can acquire transient response characteristics and enhance real-time detection efficiency.
(2) Developing new pattern recognition algorithms is a feasible way to promote the application of rapid gas sensing technology, such as the fuzzy logic and hidden Markov model. Fuzzy logic can deal with the uncertainty of sensor signals by defining fuzzy sets and rules to reduce cross-sensitivity, and it can flexibly use small sample data to determine parameters to ease the dependence on a large number of samples. It is expected that through the effective use of fuzzy sets and rules, the selectivity of target gases can be significantly improved and their dependence on large-scale data sets can be reduced. The hidden Markov model regards gas detection as a time series process and uses a probabilistic model to describe state changes, which is helpful to distinguish signals affected by cross-sensitivity and can also effectively estimate model parameters for training and verification under limited samples. The application of a hidden Markov model indicates that even in the case of limited samples, it can enhance the accuracy and robustness of gas detection through time series analysis and effectively solve the challenges brought by cross-sensitivity.

## Figures and Tables

**Table 1:** Main applications of PCA in semiconductor gas recognition.

Sensing Material	Analyte	Model	Concentration (ppm)	Operating Temperature (°C)	Train and Verify Sample Size	Accuracy	Application	Ref.
SnO_2_	C_2_H_5_OH, HCHO, C_7_H_8_, C_3_H_6_O	PCA	0.5	300	-	Successfully distinguish	Environmental monitoring	[56]
In_2_O_3_	C_3_H_6_O, C_7_H_8_, C_4_H_10_O, C_4_H_8_O	PCA	300	300	8288; 3552	88.6%	Public security	[62]
SnO_2_	C_4_H_10_O	PCA	25–150	0, 10, 20, 30, 40	-	100%	Environmental monitoring	[60]
SnO_2_	C_3_H_6_O, C_4_H_8_O, CH_3_CH_2_CH_2_OH, (CH_3_)_2_CHOH	PCA, K-NN	100	290, 250, 270, 280	-	100%	Environmental monitoring	[63]
Al_2_O_3_	NO_2_, CO	PCA	0–50	-	132, 33	94.55%	Environmental monitoring	[54]
SnO_2_	C_4_H_10_O, C_3_H_6_O, HCl, C_7_H_8_	PCA	0–300	230, 270, 250, 270	-	100%	Public security	[59]
Au, Pd, SnO_2_	CH_4_, C_3_H_8_, CO	PCA	40–200	150–500	19,975, 4994	73.6%	Environmental monitoring	[45]
SnO_2_, In_2_O_3_, WO_3_, SnO_2_, ZnO	CO, NO_2_, NH_3_, HCHO	PCA	20–60, 0.3–0.6, 1–5, 0–1.6	225	-	Successfully distinguish	Environmental monitoring	[64]
SnO_2_	CH_4_, C_2_H_5_OH, H_2_S	PCA	100, 100, 50	299.85	-	99.3%	Environmental monitoring	[55]
ZnO	C_2_H_5_OH, CH_3_CO, C_3_H_6_O, HCOOH, C_2_H_6_O	PCA	100	150–400	-	100%	Environmental monitoring	[53]
Polyether polyurethane	Different organic vapors and water vapors	PCA	0–8500	25–40	-	99%	Environmental monitoring	[65]

**Table 2:** The applications of RBFNN, ANN, DNN and BPNN algorithms in semiconductor gas identification.

Sensing Material	Analyte	Model	Concentration (ppm)	Operating Temperature (°C)	Train and Verify Sam-ple Size	Accuracy	Application	Ref.
SnO_2_	C_3_H_6_O, CH_3_CHO	ANN	5–100	300	30; -	100%	Environmental monitoring	[76]
SnO_2_	C_3_H_6_O, C_4_H_8_O, C_7_H_8_, C_4_H_10_O	K-NN	50–300	250	96; 26	100%	Public security	[61]
SnO_2_	C_3_H_6_O, C_6_H_6_, C_2_H_5_OH, HCHO, CH_3_OH, C_3_H_7_OH, C_7_H_8_	DNN_DWT, DNN_TS	200	150–225	160; 40 280; 70	98.67%, 98.33%	Environmental monitoring	[46]
SnO_2_	C_2_H_5_OH, C_3_H_6_O, CH_3_OH, CH_3_CH_2_CH_2_CH_3_, H_2_, NO_2_	CNN	≤1, 1–100	205,213	-	Relative Error: 12.3% (<1 ppm), 5.7% (>1 ppm)	Environmental monitoring	[26]
Pd-SnO_2_, Pd-WO_3_	H_2_, NH_3_, C_2_H_5_OH, C_3_H_6_O, C_7_H_8_, HCHO	pN-BPNN, K-NN	3–30	400	-	99.86%	Environmental monitoring	[11]
SnO_2_	C_7_H_8_, C_4_H_8_O, C_4_H_10_O	GUCNN-XGBoost	50–300	150–350	600; -	97.54%	Public security	[83]
MOX GSBT11, GSET21, GSDT11, GSNT11	CO, C_2_H_5_OH	ANN, DNN, 1D CNN, and 2D CNN	0–100	-	2400; 600	1D CNN > 2D CNN > DNN > ANN	Environmental monitoring	[85]
Al_2_O_3_, RuO_2_	C_2_H_5_OH, C_3_H_6_O	ANN	78–5000	-	59,268; 12,700	99.99%	Environmental monitoring	[77]
SnO_2_, In_2_O_3_	CO, CH_4_, H_2_, HCHO	BP-ANN	10–1000, 500–10,000	33–40	166; -	93.35%, 93.22%	Environmental monitoring	[12]
Al_2_O_3_	Distinguish among 11 mixtures of NO_2_ and CO	1DCNN, 2DCNN	0–50	-	106; 26	98.75%, 98.61%	Environmental monitoring	[54]
Al_2_O_3_, SnO_2_	C_3_H_6_O, C_2_H_5_OH, C_3_H_7_OH, C_4_H_9_OH	K-NN	≤3.5	350	-	91%	Environmental monitoring	[86]
E-nose	Air quality dataset	ANN	Various levels of concentra-tion	-	16,288; -	89.22%	Environmental monitoring	[87]
Eight (MOS) sensors	11 gas mixtures consisting of NO_2_ and CO gases	DCNN	20 different concentrations	-	588; -	95.2%	Environmental monitoring	[30]
E-nose (MOS sensors)	Wines with different characteristics	BPNN	-	25 ± 1	140; -	94%, 92.5%	Food industry	[80]
E-nose	C_7_H_8_, HCHO, C_6_H_6_	EQBC-RBFNN	0.1721–0.7056, 0.0668–0.1425, 0.0565–1.2856	25	396; -	96.88%	Environmental monitoring	[82]

**Table 3:** Comparison of characteristics of gas pattern recognition algorithms.

Categories	Algorithm	Property	Training Speed	Demand for Data	Robustness for Noise	Sensitive to Missing Data	Interpretability
Traditional Algorithm	PCA	Unsupervised	Fast	Low	Moderate	Low	Moderate
	LDA	Supervised	Fast	Low	Moderate	Low	Moderate
	SVM	Supervised	Moderate	Low	Low	Moderate	High
	RF	Supervised	Moderate	High	High	Moderate	High
	K-NN	Supervised	Moderate	High	High	Low	High
Neural Network Algorithm	ANN	Supervised	Moderate	Moderate	Moderate	Moderate	Low
	RNN	Supervised	Moderate	Low	High	Low	Moderate
	BPNN	Unsupervised	Slow	Moderate	Moderate	Low	High
	RBFNN	Supervised	Fast	Moderate	Moderate	Low	Moderate
	CNN	Supervised	Fast	High	High	Low	Moderate

## Abbreviations

The following abbreviations are used in this manuscript:

E-nose	Electronic-nose
MOS	Metal Oxide Semiconductor
ANN	Artificial Neural Network
CNN	Convolutional Neural Network
MLP	Multilayer Perceptron
SMO	Sequential Minimal Optimization
VOCs	Volatile Organic Compounds
DNN	Deep Neural Network
DCNN	Deep Convolutional Neural Network
DWT	Discrete Wavelet Transform
FFT	Fast Fourier Transform
R-T curve	Resistance-Temperature curve
PCF	Polynomial Curve Fitting
STFT	Short-Time Fourier Transform
SVM	Support Vector Machine
SAD	Semi-supervised Adversary-Domain Adaptive Convolutional neural network
ML	Machine Learning
PCA	Principal Component Analysis
LDA	Linear Discriminant Analysis
K-NN	K-Nearest Neighbors
SVR	Support Vector Regression
LR	Logistic Regression
RF	Random Forest
NB	Naive Bayes
ICA	Independent Component Analysis
GBDT	Gradient Boosting Decision Tree
XGBoost	Extreme Gradient Boosting
R^2^	Coefficient of Determination
RNN	Recurrent Neural Network
BPNN	Back-Propagation Neural Network
RBFNN	Radial Basis Function Neural Network
MCNA	Multi-scale Convolutional Neural Network with Attention
